# The effects of dimethyl sulfoxide and ethylene glycol as vitrification protectants on different cleavage stages of mouse embryo quality

**Published:** 2012

**Authors:** Mina Ghorbani, Rajabali Sadrkhanlou, Vahid Nejati, Abbas Ahmadi, Gholamreza Tizroo

**Affiliations:** 1 *Department of Biology, Faculty of Science, Urmia University, Urmia, Iran; *; 2 *Department of Basic Sciences, Faculty of Veterinary Medicine, Urmia University, Urmia, Iran; *; 3 *Dr. Tizroo Day Care and IVF center, Urmia, Iran.*

**Keywords:** Vitrification, Blastocyst, Morula, Ethylene glycol, Dimethyl sulfoxide

## Abstract

The effect of modified vitrification was assessed on cellular development capability in mouse embryos cultured *in vitro*. In this study, 466 embryos (from zygote to morula stages) were vitrified then thawed embryos have been incubated for in vitro farther development up to blastocyst stage. Also, vitrification and thawing procedures were the same for all experimental groups. Mouse different embryonic cleavage stages were vitrified in ethylene glycol (EG) plus dimethyl sulfoxide (DMSO) and sucrose (VS-1) and EG plus DMSO (VS-2) and thawed by directly placing the vitrified drop into sucrose solution (TS) at 37 ˚C. High recovery (72–97%) of morphologically normal embryos was evident following vitrification and thawing. Development of the vitrified morulae into blastocysts (92%) was higher (*p* < 0.05). The amount of zygote and 2-cell stages that achieved to blastocyst stage was very low. With progressing the embryo cleavage to morula stage, the embryos that reached to blastocyst were increased to its maximum number. We concluded that the modified vitrification procedure supported better survival of morula stage compared to other cleavage stages in mouse embryos.

## Introduction

Several methods have been reported for cryopreservation of mammalian oocytes and embryos.^1^^-^^9^ Vitrification is a capable of living alternative to slow freezing,^5^^,^^7^^,^^8^ with the main advantage of the elimination of ice crystal formation, the potential cause for cellular damage in slow rate freezing.^10^ According to previous studies, published reports on ultra-rapid vitrification are based on increasing cooling and warming rates. ^2^^,^^3^^,^^5^^-^^7^^,^^11^^-^^13^

During cryopreservation, the addition and removal of penetrating cryoprotective agents may create an osmotic imbalance across the cell membrane. This imbalance may cause large volumetric changes in the cells, which may alter morphology, cytoskeletal organization and function.^14^ Although excessive volumetric changes can be reduced by adding and removing cryoprotectants in a step wise fashion, prolonged exposure of cells to cryoprotectants at non-freezing temperature may induce toxic effects.^15^^,^^16^ Fast cooling and thawing during ultra-rapid vitrification reduces the time of exposure of cells to cryoprotectants at non-freezing temperature and may reduce toxic stress to oocytes and embryos. Moreover, ultra-rapid vitrification and thawing also minimize chilling injury, as the cells are exposed to critical temperature zones for comparatively short interval.^5^ Therefore, good survival and development of oocytes and embryos could be achieved.^5^^,^^17^

Various methods of ultra-rapid vitrification of mammalian oocytes and embryos have been used including vitrification in droplets,^2^^,^^12^^,^^13^ on electron microscope grids,^3^ in open pulled straws,^5^ in a cryoloop,^6^ on solid surface,^7^ and on cryotop.^8^ In this study we used the open pull straw for vitrification procedure to determine the best stage in development stages of mouse embryos for vitrification and their cryopreservation cultured *in vitro*.

## Materials and Methods

**Animals. **Mature female NMRI mice (6 to 10 weeks old) were induced to superovulate with intraperitoneal injections of 7.5 IU PMSG1 (Intervet, Boxmeer, The Netherlands) and 7.5 IU hCG (Intervet, Boxmeer, The Netherlands) given 48 hr apart. Thirteen hr after hCG injection the mice were euthanized by cervical dislocation and ovulated unfertilized oocytes and sperms were collected from females and males. After IVF in the culture medium (HTF solution consisted of 4 mg mL^-1 ^BSA), embryos at various development stages (1-cell, 2-cell, 4-cell, 8-cell, and Morula) were vitrified using the VS-1 and VS-2 media.

**Vitrification of embryos. **Embryos were vitrified in cryoprotectants solution (VS) in 0.25 mL open pull straws based on the method described by Kasai *et al*.^18^ Briefly, one to three embryos were loaded in a straw. Embryos were transferred into the larger column of vitrification solution in straw for exposing to VS-1 for 4 minutes and VS-2 for 40 sec. Then the straw was placed horizontally in liquid nitrogen and immersed into it. The handling of vitrified embryos during all manipulations was performed at room temperature until immersed in the freezing medium. After warming, the embryos were kept at 25 ˚C until placed in the 5% CO_2_ incubator. All procedures were conducted in room at 25 ˚C.

**Fig. 1:**
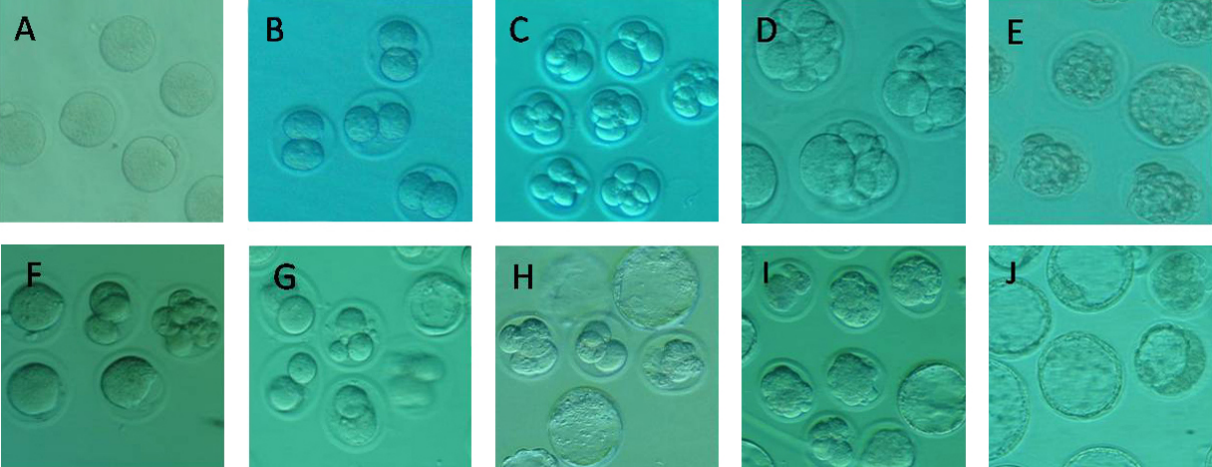
Different cleavage stages of fresh mouse embryo in the culture medium (HTF solution consisted of 4 mg mL^-1 ^BSA); **A.** Zygote; **B.** 2-cell embryo; **C.** 4-cell embryo; **D.** 8-cell embryo; **E.** Morula (60×).

**Thawing. **After 2 weeks of storage in liquid nitrogen, embryos were re-warmed for *in vitro* culture. For re-warming, straws containing the embryos were held at ambient temperature for 10 Sec after removal from the liquid nitrogen. The narrow end was immersed vertically in a well containing TS-1 at 37 ˚C. After 1 minute embryos were transferred into TS-2 with the same medium for 3 minutes. The embryos were allocated in TS-3 for 3 min (Fig. 1).

**Culture. **Vitrified/warmed embryos were cultured in 5% CO_2_ in air in a humid chamber at 38.5 ˚C. One to three embryos were vitrified in one straw and cultured after warming in 50 mL droplets (for not more than 6 h) or in 500 mL (for long periods) of the culture medium (HTF solution consisted of 4 mg mL^-1 ^BSA) in culture dishes with covering oil. The embryos were regularly evaluated between 6 and 14 h of culture (Fig. 1).

**Statistical analyses.** Data were analyzed using the two proportion test of Minitab (version 16, Minitab Inc., Pennsylvania, USA) software and differences were considered significant at *p* < 0.05.

Different cleavage stages of vitrified mouse embryo after thawing in the culture medium until reaching to blastocyst; **F.** Zygote; **G.** 2-cell embryo; **H.** 4-cell embryo; **I.** 8-cell embryo;** J. **Morula (60×).

## Results

In this study, 466 embryos (from zygote to morula stages) were vitrified then thawed embryos have been incubated for *in vitro* farther development up to blastocyst stage. In this experiment vitrification and thawing procedures were the same for all experimental groups. Following thawing the majority of vitrified embryos had morphologically normal shape (Table 1). Cleavage rate reaching to the blastocyst stage was correlated with the rate of embryo development. The amount of zygote and 2-cell stages that achieved to blastocyst stage was very low. With progressing the embryo cleavage to morula stage, the embryos that reached to blastocyst were increased to its maximum number.

**Table 1:** *In vitro* development of various stages of mouse embryos after vitrification

**Developmental ** ** stages**	**Vitrified embryos (No.)**	**Recovered embryos ** **(%)**	**Morphologically normal ** **(%)**	**Cleavages** **(%)**	**Developed to blastocyst ** **(%)**
**zygote**	89	92	72	37	16
**2-Cell**	119	92	86^a^	42	25
**4-Cell**	79	96	90^a^	89^a^^b^	71^a^^b^
**8-Cell**	86	98^a^	93^a^	91^a^^b^	80^a^^b^
**Morula**	93	100^a^	97^a^^b^^c^	96^a^^b^	92^a^^b^^c^^d^

Values with the same superscripts are significantly different (*p* < 0.05).

a Significant difference of zygote data with the other developmental stages data.

b Significant difference of 2-cell stage data with the other developmental stages data.

c Significant difference of 4-cell stage data with the other developmental stages data.

d Significant difference of 8-cell stage data with the other developmental stages data.

As shown in Tables 1 and 2, results for higher levels of developmental stages are improved from zygote and 2-cell stages to morula embryos, respectively.

Eighty two out of 89 (92%) of vitrified zygotes were recovered, however, only 59/82 (72%) of recovered zygotes were morphologically normal and the rest of them were destroyed (23/82, 28%) (Table 1 and 2). Among morphologically normal zygotes, only 22/59 (37%) were developed to cleavage stage, 9/59 (16%) reached to blastocyst and 50/59 (84%) were arrested.

In vitrified 2-cell embryos, 110/119 (92%) of them were recovered following thawing, 95/110 (86%) of them were morphologically normal and the rest of recovered embryos were destroyed (15/110, 14%) (Tables 1 and 2). Among morphologically normal 2-cell embryos, 40/95 (42%) were developed to cleavage stage, 24/95 (25%) became blastocyst and 71/95 (75%) were arrested.

In the vitrified 4-cell embryos, 76/79 (96%) were recovered, 68/76 (90%) of this group were morphologically normal and the rest of them were destroyed (8/76, 10%). Among morphologically normal 4-cell embryos61/68 (89%) were developed to cleavage stage, 48/68 (71%) reached to blastocyst and 20/68 (29%) were arrested.

In the vitrified 8-cell embryos, 85/86 (98%) were recovered, 79/85 (93%) of recovered embryos were morphologically normal and the rest of them were lysed (6/85, 7%). Among morphologically normal 8-cell embryos, 72/79 (91%) were developed to cleavage stage, 63/79 (80%) became blastocyst and 16/79 (20%) were arrested.

In vitrified morulae, 93/93 (100%) were recovered, 91/93 (97%) of this group were morphologically normal and the rest of them were destroyed (2/93, 3%). Among morphologically normal morulae, 87/91 (96%) were developed to farther cleavage stage, 84/91 (92%) reached to blastocyst and 7/91 (8%) were arrested.

**Table 2:** Percentages of lyses and arrested embryos after thawing of various developmental stages *in vitro*.

**Developmental ** **stages **	**Vitrified embryos** ** (No.)**	**Lyses** **(%)**	**Arrested** **(%)**	**Type 1** **(%)**	**Type 2** **(%)**	**Type 3** **(%)**
**zygote**	89	28	84	37	6	41
**2-Cell**	119	14^a^	75	16^a^	28^a^	31
**4-Cell**	79	10^a^	29^a^^b^	2^a^^b^	5	22^a^^b^
**8-Cell**	86	7^a^	20^a^^b^	3^a^	4	13^b^
**Morula**	93	3^a^^b^^c^	8^a^^b^^c^^d^	1^a^	2	5

Values with the same superscripts are significantly different (*p* < 0.05).

a Significant difference of zygote data with the other developmental stages data.

b Significant difference of 2-cell stage data with the other developmental stages data.

c Significant difference of 4-cell stage data with the other developmental stages data.

d Significant difference of 8-cell stage data with the other developmental stages data.

## Discussion

Vitrification is a potential alternative to traditional slow-rate freezing for preserving oocytes and embryos in various species. The unique advantage of vitrification is elimination of mechanical injury caused by intra- or extra-cellular ice crystal formation and reduction of chilling injury by shortening duration of exposure of cell to critical temperature point. The concept of ultra-rapid vitrification has been emerged in recent years.^2^^,^^3^^,^^9^^,^^12^^,^^13^^,^^19^^-^^21^

An ultra-rapid cooling rate during vitrification can practically be achieved either by minimizing the volume of solution to be vitrified or by making direct contact between vitrification solution and liquid nitrogen otherwise through the combination of the procedures. The major disadvantages of the previous vitrification protocols were the relatively large volume of the vitrified drop and the delay before the drop floating on the surface of liquid nitrogen which probably reduced the actual cooling rate.^2^^,^^12^^,^^13^^,^^19^^,^^22^

In our work we eliminated these disadvantages and tried to achieve a higher cooling rate reducing the volume of vitrified drop and immersing in liquid nitrogen immediately onto the surface of the liquid nitrogen (which helped the drop to quickly vitrify and sink in liquid nitrogen). In addition we attempted to reduce cytotoxicity through shortening the total duration of exposure of embryos to cryoprotectants and using an EG-based vitrification solution. It was reported previously that EG had low cytotoxicity for embryos.^23^^,^^24^

Several permeating cryoprotectants have been used for cryopreservation of embryos and resulted in successful production of young. Among the cryoprotectants, DMSO has most frequently been used both in slow freezing^25^^-^^28^ and vitrification procedures .^29^^,^^30^

Our method ensured a rapid thawing rate of vitrified embryos by directly placing the vitrified drop into sucrose solution at 37 ˚C. As expected, we achieved high developmental competence of the vitrified embryos. The aim of the present study was to compare survival rates of mouse embryos cryopreserved at various developmental stages. For the cryopreservation, embryos were vitrified using VS, an EG-based solution. This vitrification solution was foremost developed for mouse embryos as a low-toxicity solution.^18^

Mouse embryos at various developmental stages were vitrified by a 2-step method in which embryos were directly suspended in vitrification solution before cooling. When survival was assessed in culture, it was shown that high proportions (92%) of morulae retained the ability to develop to the blastocyst stage (Table 1). Major mechanisms of cell injury in vitrified embryos would be cryoprotectant toxicity, intracellular ice forming and osmotic stress during removal of permeated cryoprotectant. More permeation will be favorable to prevent intracellular ice forming but not cryoprotectant toxicity and osmotic stress. Upon suspension in VS-1 or VS-2, embryos at any stage shrink rapidly and considerably, and are remained shrunken, because VS solutions contain not only a permeating agent (i.e. ethylene glycol) but also nonpermeating sugar (i.e. sucrose). So, it was not possible to estimate how much EG had been permeated during suspension from the volume change of the embryos. However, zygote and 2-cell embryos appear to be less permeable to EG than 4- and 8-cell embryos and morulae, because the latter embryos survived vitrification after 2-step exposure to VS-2 for only 40 sec. Although insufficient permeation after 2-step treatment might cause intracellular ice formation, its influence was not apparent in the morphology of vitrified embryos, since most of the recovered embryos appeared normal.

In conclusion, the morula would be the preferred stage for mouse embryo cryopreservation, because the survival rate of vitrified morulae was 97% as assessed by morphology or by in vitro development up to blastocyst stage.
